# Opioid Toxicity Associated With Tirzepatide in a Patient on Chronic Oral Morphine

**DOI:** 10.7759/cureus.115380

**Published:** 2026-08-28

**Authors:** Nirosh E Mataraarachchi, Oscar D Dellinger, Ala Babakhanians

**Affiliations:** 1 Medicine, University of California, Los Angeles, Los Angeles, USA; 2 Geriatrics, VA Greater Los Angeles Healthcare System, Los Angeles, USA; 3 Pharmacology, VA Greater Los Angeles Healthcare System, Los Angeles, USA

**Keywords:** acute kidney injury, adverse effects of glp-1 agonists, chronic opioid use, opioid use in chronic kidney disease, opioid use in pain management

## Abstract

Tirzepatide, a dual glucose-dependent insulinotropic polypeptide/glucagon-like peptide-1 (GLP-1) receptor agonist, delays gastric emptying and can alter the pharmacokinetics of coadministered oral medications, particularly those with narrow therapeutic indices. We report the first case of life-threatening opioid toxicity precipitated by tirzepatide initiation in a patient on chronic oral sustained-action opioid therapy. A 53-year-old man with primary progressive multiple sclerosis complicated by quadriplegia, maintained on oral sustained-action morphine sulfate 180 mg/day for 13 years without incident, presented with opioid-induced respiratory failure six weeks after starting tirzepatide 2.5 mg weekly (escalated to 5 mg at week 4). He required 12 mg of intranasal naloxone for reversal. Concurrent findings included acute kidney injury (creatinine 1.04 mg/dL from a baseline of 0.5-0.6 mg/dL), hemoconcentration, metabolic acidosis, and ketonuria consistent with tirzepatide-associated dehydration. His caregiver had prospectively noted recurrent somnolence after each weekly injection. Two complementary mechanisms likely converged: tirzepatide-mediated delayed gastric emptying disrupting the release profile of sustained-action morphine and dehydration-induced kidney injury impairing renal clearance of morphine-6-glucuronide, the active opioid metabolite. Concurrent use of other renally cleared CNS depressants likely amplified toxicity. A prior hospitalization for sepsis with similar renal impairment, before tirzepatide initiation, did not produce opioid toxicity, offering a useful, though imperfect, point of comparison. Morphine was reduced to 60 mg/day; at follow-up, the patient reported equivalent pain control on one-third of his prior dose. This case highlights a previously unrecognized, clinically significant drug interaction with urgent implications as GLP-1-based therapies are increasingly co-prescribed with chronic opioid therapy, underscoring the need for clinician vigilance and formal pharmacokinetic study.

## Introduction

The rapid expansion of glucagon-like peptide-1 (GLP-1) receptor agonists and dual glucose-dependent insulinotropic polypeptide (GIP)/GLP-1 receptor agonists into clinical practice represents one of the most significant shifts in obesity and metabolic medicine in recent decades [1]. Tirzepatide (Zepbound^®^/Mounjaro^®^; Eli Lilly and Company, Indianapolis, IN, USA), a novel dual GIP and GLP-1 receptor agonist, has demonstrated substantial efficacy for weight reduction and glycemic control [2], leading to rapidly increasing prescribing across primary care, endocrinology, and obesity medicine. As of 2025, GLP-1-based therapies represent one of the fastest-growing drug classes in the United States, with prescription volume increasing markedly year over year [1].

A pharmacologic consequence of GLP-1 receptor agonism is delayed gastric emptying, mediated through GLP-1- and GIP-receptor-dependent inhibition of antral motility and pyloric contraction [3,4]. This effect is central to the drugs’' weight-loss mechanism, since slowing nutrient transit promotes satiety and reduces caloric intake, but it also has the potential to disrupt the absorption pharmacokinetics of concomitantly administered oral medications. The tirzepatide prescribing information explicitly advises caution when coadministering drugs “dependent on threshold concentrations for efficacy” or drugs with a “narrow therapeutic index” and reports that a single 5 mg dose reduced acetaminophen maximum concentration (Cmax) by 55% and delayed time to maximum concentration (Tmax) by one hour [5].

Sustained-action (extended-release) oral formulations are engineered with precise release kinetics that depend on predictable GI transit times. Perturbation of gastric emptying has the potential to fundamentally alter the pharmacokinetic profile of such formulations, with consequences for both drug efficacy and toxicity. This risk is most clinically significant for medications with narrow therapeutic indices, a category that prominently includes opioid analgesics. Physiologically based pharmacokinetic (PBPK) modeling has predicted area under the concentration-time curve (AUC) increases of 64-205% for certain oral medications coadministered with GLP-1 receptor agonists [6]. A systematic review of GLP-1 receptor agonist drug interactions explicitly cautioned that reassuring pharmacokinetic data from healthy volunteer studies “should be carefully generalized to cases of background kidney dysfunction or when using drugs with narrow therapeutic index” [7].

Chronic opioid therapy is prevalent among patients with significant disability, cancer-related pain, and chronic non-cancer pain conditions, and obesity is overrepresented in these populations. As GLP-1-based therapies expand into primary care and obesity medicine, coprescription with chronic opioids will become increasingly common. However, to date, no pharmacokinetic studies have evaluated the interaction between GLP-1 receptor agonists and oral opioid formulations, and no cases of GLP-1 receptor agonist-associated opioid toxicity in patients on chronic prescribed opioid therapy have been reported in the literature.

We report the first case of life-threatening opioid toxicity precipitated by tirzepatide initiation in a patient maintained on an unchanged oral sustained-action morphine regimen for 13 years. This case delineates the dual pharmacokinetic mechanisms involved, considers a comparative reference point from a prior hospitalization, and highlights a critical gap in the evidence base as this drug class expands into vulnerable patient populations. This case report was prepared in accordance with the CAse REport (CARE) guidelines [8].

## Case presentation

A 53-year-old man with primary progressive multiple sclerosis complicated by C4-level quadriplegia, severe generalized spasticity, and chronic neuropathic and musculoskeletal pain was brought to the ED after being found unarousable by his wife. He was a veteran receiving care through the VA Greater Los Angeles Healthcare System’s Home-Based Primary Care (HBPC) program.

The patient had been maintained on oral sustained-action morphine sulfate 45 mg every six hours (180 mg/day) for 13 consecutive years without any dose adjustments, episodes of toxicity, or concerns regarding adherence. His wife served as his sole caregiver and medication manager and was independently described by multiple providers as meticulous about correct dosing. His additional stable medications included tizanidine 10 mg four times daily (40 mg/day), pregabalin 100 mg four times daily (400 mg/day), baclofen 20 mg four times daily (80 mg/day), trazodone 50 mg at bedtime as needed, and alprazolam 0.25 mg as needed (used approximately two to three tablets per month). Baseline serum creatinine was chronically low, consistent with reduced muscle mass from quadriplegia.

The patient’s clinical stability over the preceding 13 years was well-documented. Of particular relevance, approximately 14 months before the current admission, he was hospitalized for right lower extremity cellulitis with sepsis, with tachycardia, leukocytosis, an elevated lactate, and a mildly elevated creatinine during the admission. Despite this physiologic stress and comparable renal impairment, he did not develop opioid toxicity and was discharged on his full, unchanged home morphine regimen. Tirzepatide had not yet been initiated at the time of that admission.

Six weeks prior to the current presentation, tirzepatide (Zepbound^®^) 2.5 mg subcutaneously weekly was initiated by the HBPC team for weight management (BMI 33 kg/m²). After four weeks, the dose was escalated to 5 mg weekly per standard titration protocol. No changes were made to any other medications throughout this period. Beginning with the first injection, the patient’s wife noted that he “tends to become sleepy when he takes Zepbound,” a recurring pattern of somnolence temporally linked to each weekly injection. This observation was documented by the admitting team independently before any hypothesis regarding a drug interaction had been formulated.

Three days after his most recent tirzepatide injection (the second injection at the 5 mg dose level), the patient experienced progressive lethargy and decreased oral intake over 48 to 72 hours, culminating in unresponsiveness. On arrival to the ED, he had pinpoint pupils, central cyanosis, and bradypnea. He received intranasal naloxone 4 mg, with a partial response; a total of 12 mg of intranasal naloxone was required to achieve full reversal of respiratory depression and restoration of consciousness, a response pattern consistent with opioid toxicity from a long-acting opioid with sustained tissue concentrations.

Admission laboratory studies revealed an acute kidney injury (AKI) with an approximately 2-fold increase in creatinine from his low baseline, an elevated blood urea nitrogen (BUN), a metabolic acidosis with a decreased venous pH and decreased bicarbonate, hemoconcentration with an elevated hemoglobin relative to his baseline, positive urine ketones, and an elevated high-sensitivity troponin T (Table 1). WBC count was also elevated, consistent with a stress leukocytosis. CT of the head (Figure 1) and MRI of the brain, including diffusion-weighted and apparent diffusion coefficient sequences (Figure 2), showed no acute intracranial findings. Serum morphine and morphine-6-glucuronide (M6G) levels were not obtained during the acute presentation; consequently, the proposed increase in opioid exposure and M6G accumulation discussed below could not be directly demonstrated in this patient.

**Table 1:** Laboratory findings on presentation. Laboratory findings on presentation, with a comparator column from the prior hospitalization approximately 14 months earlier and reference ranges. Presenting findings were consistent with AKI, dehydration-related hemoconcentration, and metabolic acidosis; prior findings were consistent with sepsis without opioid toxicity. AKI, acute kidney injury; BUN, blood urea nitrogen

Laboratory test	Presenting value	Value 14 months prior	Reference range	Interpretation
Serum creatinine	1.04 mg/dL	0.70-0.85 mg/dL (trend)	0.7-1.2 mg/dL	Presenting: within range, but ~2-fold increase from patient’s low baseline (0.5-0.6 mg/dL); consistent with AKI. Prior: mildly elevated, resolved without opioid toxicity
BUN	26 mg/dL	Not obtained	6-20 mg/dL	Presenting: elevated
Venous pH	7.32	Not obtained	7.35-7.45	Presenting: decreased (acidemia)
Serum bicarbonate	14 mmol/L	Not obtained	22-29 mmol/L	Presenting: decreased (metabolic acidosis)
Hemoglobin	16.0 g/dL	Not obtained	13.3-17.7 g/dL	Presenting: within range, but increased from baseline (14.8 g/dL); consistent with hemoconcentration
Urine ketones	20 mg/dL	Not obtained	Negative	Presenting: positive; consistent with dehydration
High-sensitivity troponin T	69 ng/L	Not obtained	<22 ng/L (male)	Presenting: elevated
WBC count	12.39 × 10³/µL	20.71 × 10³/µL	4.5-11.0 × 10³/µL	Presenting: elevated. Prior: elevated, consistent with sepsis
Lactate	Not obtained	3.93 mmol/L	0.5-1.6 mmol/L	Prior: elevated, consistent with sepsis

**Figure 1:**
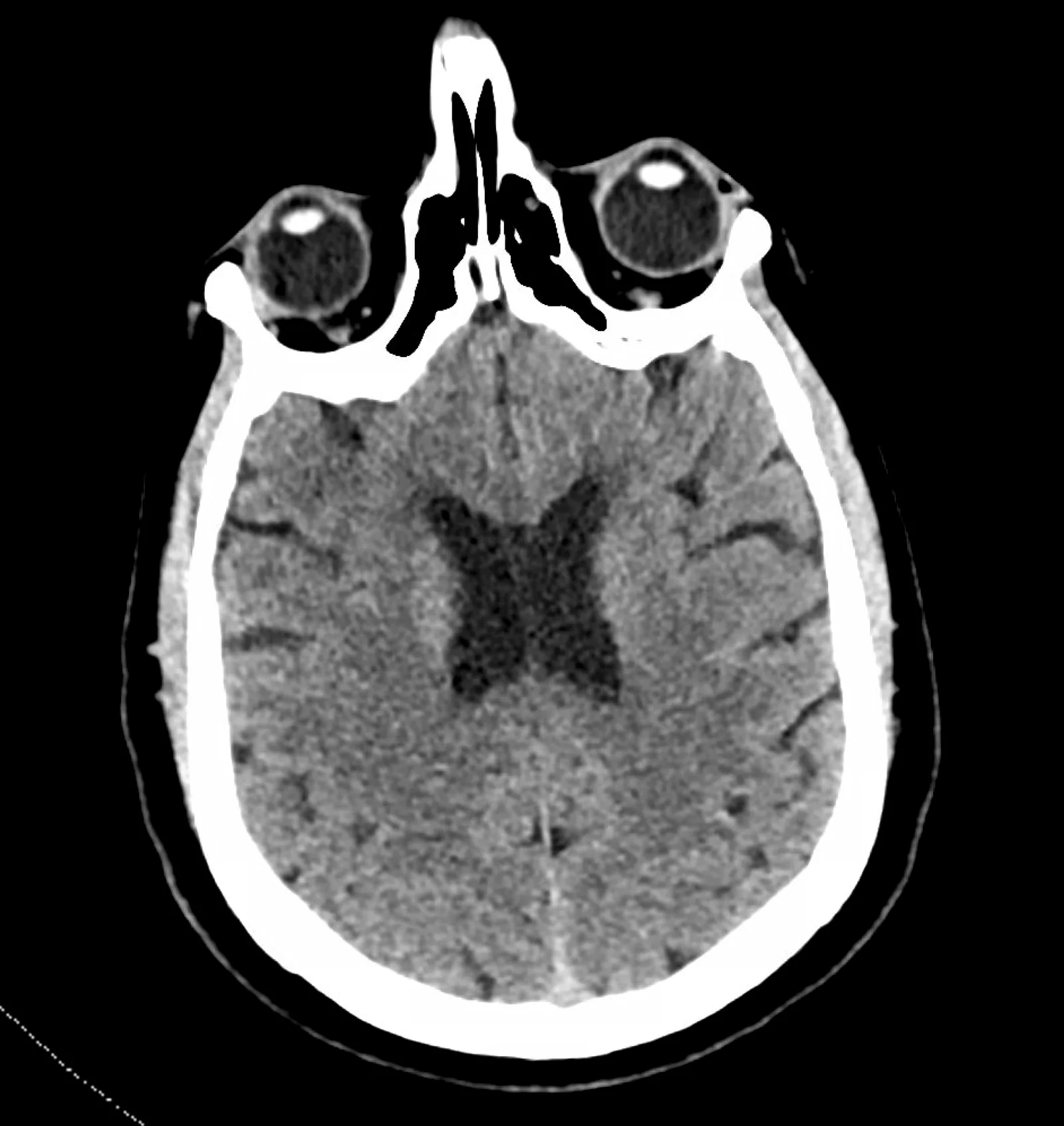
Noncontrast axial CT of the head at initial presentation demonstrating no acute intracranial hemorrhage, mass effect, or midline shift.

**Figure 2:**
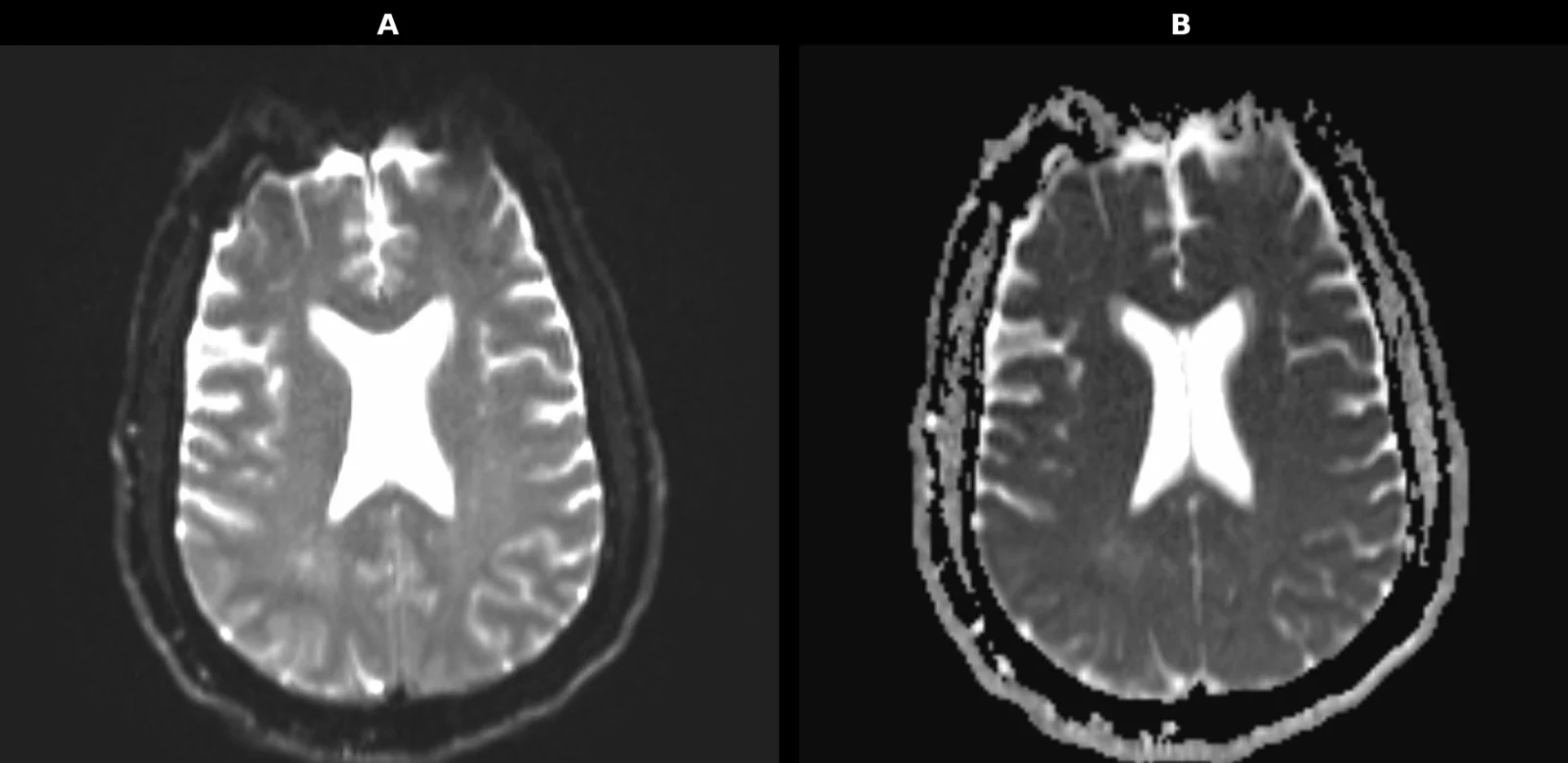
Axial brain MRI at initial presentation. Axial diffusion-weighted imaging (A) and corresponding apparent diffusion coefficient map (B) of the brain showing no area of restricted diffusion to suggest an acute infarct.

The constellation of hemoconcentration, ketonuria, and metabolic acidosis in the absence of infectious or other precipitants was consistent with prolonged reduced oral intake and volume depletion, both recognized effects of GLP-1 receptor agonist therapy. No changes in opioid dosing, medication adherence, pill count, or other medications had occurred in the weeks preceding presentation.

AKI resolved with IV fluid resuscitation; creatinine normalized to within his low baseline range by hospital day 3. Tirzepatide was permanently discontinued. Morphine was reduced to 15 mg every six hours (60 mg/day). Pregabalin was also reduced from 100 mg to 50 mg four times daily during the admission, reflecting clinical recognition of the polypharmacy contribution to CNS depression in the setting of renal impairment.

At follow-up 10 days after hospital discharge, the patient reported equivalent pain control on 60 mg/day, one-third of his 13-year stable dose of 180 mg/day, and did not wish to increase his morphine dose. This observation persisted at subsequent follow-up.

## Discussion

This case documents the first reported instance of life-threatening opioid toxicity precipitated by tirzepatide initiation in a patient on chronic oral sustained-action opioid therapy. It illustrates a pharmacokinetic drug interaction operating through two complementary, interdependent mechanisms and provides supportive, though not definitive, evidence of a causal relationship through the patient’s 13-year regimen stability, the caregiver’s prospective temporal observations, and a comparative reference point afforded by a prior hospitalization under a different, though also physiologically stressful, clinical circumstance without tirzepatide exposure. This comparison is discussed further below, along with its important limitations.

Two mechanistically distinct but functionally related pharmacokinetic processes likely converged to produce opioid toxicity in this patient. In simpler terms, tirzepatide can slow how quickly the stomach empties, which may cause a sustained-release medication like this patient's morphine to be absorbed unevenly and in larger-than-intended amounts; separately, if tirzepatide causes dehydration severe enough to stress the kidneys, the body may become less able to clear an active breakdown product of morphine (M6G) from the blood, allowing it to build up. Both processes are discussed in detail below.

Mechanism 1: altered GI absorption

Tirzepatide delays gastric emptying through GLP-1 and GIP receptor-mediated inhibition of antral motility and pyloric contraction [3,4]. The Zepbound prescribing information reports that a single 5 mg dose reduced acetaminophen Cmax by 55% and delayed Tmax by one hour, with the effect most pronounced after the first dose at each new dose level [5]. PBPK modeling by Hooper et al. predicted AUC increases of 64% to 205% for certain oral medications co-administered with GLP-1 receptor agonists [6].

Sustained-action morphine formulations are engineered to release drug over a predictable six-hour window, depending on normal GI transit. Delayed gastric emptying could disrupt this release profile through several mechanisms: (1) prolonged gastric retention may alter pH-dependent dissolution of the sustained-release matrix; (2) delayed transit to the small intestine, the primary site of absorption, may cause temporal overlap between sequential doses administered every six hours, producing progressive dose-stacking; and (3) altered gastric residence time may change the ratio of morphine released in the stomach versus the small intestine, affecting first-pass hepatic metabolism and overall bioavailability. The cumulative effect over days to weeks of tirzepatide exposure could progressively increase effective drug exposure without any change in prescribed dose.

Mechanism 2: impaired renal clearance of M6G

Morphine undergoes hepatic glucuronidation by UDP-glucuronosyltransferase 2B7 to produce morphine-3-glucuronide (M3G, approximately 60% of metabolites) and M6G (approximately 10-15%) [9,10]. M6G is pharmacologically active with analgesic and respiratory-depressant potency estimated at two to four times that of the parent compound, and both glucuronide metabolites are renally excreted [10]. In renal failure, M6G accumulates and crosses the blood-brain barrier, producing prolonged CNS and respiratory depression. D’Honneur et al. demonstrated that cerebrospinal fluid M6G concentrations at 24 hours in patients with renal failure were 15 times greater than in patients with normal renal function following a single oral morphine dose [9]. The morphine prescribing information warns that “M3G and M6G may accumulate to much higher plasma levels in patients with renal failure” [10].

This patient’s admission creatinine of 1.04 mg/dL represented an approximately 2-fold increase from his unusually low baseline (0.5-0.6 mg/dL), reflecting his reduced muscle mass from quadriplegia. While the absolute creatinine value falls within the laboratory reference range, the proportional increase indicates a clinically significant reduction in glomerular filtration rate for this individual. The concurrent hemoconcentration, elevated BUN, and urine ketones were consistent with GLP-1 receptor agonist-associated reduced oral intake and volume depletion, which is explicitly warned against in the tirzepatide prescribing information as a risk factor for AKI. AKI resolved promptly with fluid resuscitation, confirming a pre-renal etiology attributable to dehydration.

Critically, these two mechanisms are not independent: the dehydration causing AKI is itself a downstream pharmacologic effect of tirzepatide-associated appetite suppression and nausea, making the entire causal chain, from delayed gastric emptying to M6G accumulation, attributable to tirzepatide initiation.

Causal assessment

Several convergent features establish a strong causal signal. The patient had been on an identical morphine regimen for 13 consecutive years without incident. This extraordinary period of stability substantially reduces the prior probability of a spontaneous overdose event unrelated to a new medication. The Naranjo Adverse Drug Reaction Probability Scale yielded a score of 4 (“possible”), though this instrument likely underestimates the true causal probability in this case [11]. The Naranjo scale does not account for the extraordinary regimen stability, the caregiver’s prospective temporal observations, or the mechanistic implausibility of a spontaneous event after 13 years. The scale’s deduction of a point for “alternative causes” (the AKI) is mechanistically inappropriate here because the AKI is most plausibly a downstream consequence of tirzepatide-induced dehydration rather than an independent cause.

Comparative evidence from prior hospitalization

The prior hospitalization provides a potentially informative, though methodologically imperfect, point of comparison and should not be interpreted as a controlled comparison. During that earlier admission for cellulitis with sepsis, the patient had comparable renal impairment (creatinine 0.70-0.85 mg/dL), was receiving the same morphine regimen, and did not develop opioid toxicity; he was discharged on his full home morphine dose. However, the two episodes differed in several important respects beyond the presence or absence of tirzepatide: the underlying illness differed (sepsis versus opioid toxicity), the trajectory and etiology of renal impairment differed, hydration status was managed differently, and medication administration differed, since the prior admission involved missed morphine doses requiring bridging with as-needed IV morphine. These differences mean the two admissions cannot be equated as a true natural experiment, and the comparison should be interpreted as one contextual observation among several, rather than as confirmatory evidence.

Temporal association and caregiver observations

The caregiver’s independent observation of recurrent somnolence with each weekly tirzepatide injection, documented before any drug interaction hypothesis was proposed, provides a prospective temporal signal that the Naranjo Scale and most causality assessments cannot adequately capture. This recurring weekly pattern is consistent with the pharmacokinetic model: each injection produces delayed gastric emptying most pronounced in the days following administration, with somnolence representing a subclinical manifestation of the same process that eventually reached a toxic threshold at week 6.

Contribution of concomitant CNS depressants

This patient was simultaneously maintained on multiple oral CNS depressants, including tizanidine, pregabalin, baclofen, and intermittent trazodone and alprazolam, all orally administered and all theoretically subject to the same tirzepatide-mediated gastric emptying delay. Pregabalin is of particular pharmacokinetic concern because it is predominantly renally excreted unchanged; therefore, the same AKI that impaired M6G clearance would also reduce pregabalin clearance and increase its plasma concentrations [12]. This was clinically recognized by the admitting team, who reduced pregabalin from 100 mg to 50 mg four times daily at admission alongside morphine reduction.

Baclofen, which can cause respiratory depression and CNS depression, particularly in the setting of renal impairment, also undergoes predominantly renal excretion. Tizanidine, though primarily hepatically metabolized, is also a CNS depressant that could have contributed independently. These agents should be considered not merely as amplifiers of a tirzepatide-morphine interaction but as a genuine competing explanation: cumulative CNS depressant burden and pregabalin or baclofen accumulation from AKI alone, independent of any change in morphine pharmacokinetics, could plausibly account for some or all of the observed sedation and respiratory depression, and this case cannot determine which contributor predominated. Simultaneous pharmacokinetic perturbation of multiple CNS depressants through a single mechanism (tirzepatide-induced gastric emptying delay and AKI) could also produce additive or synergistic toxicity effects not predictable from studies of individual drug interactions. This polypharmacy context is particularly relevant because many patients on chronic opioid therapy for spasticity, disability-related pain, or chronic non-cancer pain are co-prescribed gabapentinoids and muscle relaxants, precisely the population increasingly being referred for GLP-1-based obesity treatment.

Reduced morphine requirement and possible mechanisms

A clinically striking observation is that the patient achieved equivalent pain control at 60 mg/day after 13 years on 180 mg/day, a difference that persisted at follow-up and was not desired to be reversed by the patient. Several mechanisms may contribute. Opioid-induced hyperalgesia, a paradoxical increase in pain sensitivity from chronic high-dose opioid exposure through central sensitization, may have been partially reversed by the forced dose reduction. Alternatively, opioid receptor tolerance reset following the acute event and naloxone administration may have allowed lower doses to achieve equivalent receptor occupancy and analgesia.

There is also emerging evidence that GLP-1 receptor agonists may modulate opioid sensitivity pathways through CNS mechanisms. Population-level studies have found GLP-1 receptor agonists associated with a 42% to 68% lower risk of opioid overdose in patients with opioid use disorder, and preclinical data demonstrate GLP-1 receptor-mediated attenuation of opioid reward-seeking behavior [13]. Whether residual GLP-1 receptor-mediated modulation of opioid sensitivity from prior tirzepatide exposure contributed to sustained efficacy at a reduced dose remains speculative but is consistent with broader evidence on this drug class and its interactions with opioid neurobiology [14].

Clinical implications

Several clinical implications follow from this case. Clinicians should systematically screen for chronic opioid use, particularly sustained-action and extended-release formulations, before initiating GLP-1-based therapy, recognizing that this population carries substantially elevated pharmacokinetic risk. Patients and caregivers should be explicitly counseled to monitor for increased sedation, confusion, or respiratory changes during GLP-1 receptor agonist initiation and dose escalation, which represent the highest-risk windows for gastric emptying disruption. Proactive opioid dose reduction or formulation change, for example, transition to immediate-release preparations with scheduled dosing, should be considered before or concurrent with GLP-1 receptor agonist initiation in high-risk patients. Renal function and hydration status should be monitored during the initiation period, particularly in patients on renally cleared co-medications, consistent with the tirzepatide prescribing information’s own caution regarding volume depletion [5]. Take-home naloxone should be ensured for all patients on chronic opioid therapy who are initiated on a GLP-1-based agent, in line with the FDA’s recommendation to co-prescribe naloxone for patients at increased risk of opioid overdose, including those on concurrent CNS depressants [15], and caregivers should be trained in its use.

Pharmacovigilance implications

From a pharmacovigilance standpoint, this case underscores a critical gap in the evidence base. Existing pharmacokinetic studies of GLP-1 receptor agonist drug interactions were conducted in healthy volunteers using drugs with wide therapeutic indices, populations, and compounds that may not generalize to patients with baseline renal vulnerability, complex polypharmacy, and narrow-therapeutic-index medications taken at high chronic doses [7]. This case has been reported to the FDA MedWatch pharmacovigilance system, and we urge clinicians who observe similar events to report them, as accumulation of case-level data will be essential to characterize the prevalence and severity of this interaction.

Limitations

This case has inherent limitations of a single case report. Serum morphine and M6G levels were not obtained during the acute presentation; therefore, the proposed increase in opioid exposure and M6G accumulation could not be directly demonstrated, and the pharmacokinetic mechanisms proposed here remain plausible hypotheses rather than confirmed findings. The same absence of drug-level data limits any distinction between opioid metabolite accumulation and the competing contribution of concurrent CNS depressants discussed above, as well as between the relative contributions of altered GI absorption versus impaired renal clearance. The Naranjo score of “possible” reflects both the structural limitations of the instrument and genuine uncertainty about causal attribution in this case. These limitations mean the temporal association, the mechanistic plausibility, and the comparative context provided by the prior hospitalization should be viewed as supportive of an association rather than as proof of causation, and they underscore the need for formal pharmacokinetic studies.

## Conclusions

This case documents the first reported instance of life-threatening opioid toxicity precipitated by tirzepatide initiation in a patient maintained on chronic oral sustained-action morphine therapy for 13 years, through two convergent pharmacokinetic mechanisms: delayed gastric emptying causing sustained-release dose-stacking and dehydration-induced AKI impairing clearance of the active opioid metabolite M6G. The patient’s regimen stability, prospective caregiver observations, and a comparative reference point from a prior hospitalization together support a plausible causal association, though the contribution of concurrent CNS depressants cannot be excluded and causality cannot be definitively established from a single case without confirmatory drug-level data.

As GLP-1-based therapies are prescribed to an expanding population that also receives chronic opioid therapy, this interaction carries substantial population-level safety implications. Clinicians should screen for chronic opioid use before initiating GLP-1-based therapy, and formal pharmacokinetic studies in clinically representative patients are urgently needed.
